# Letter to the editor: importance of a careful investigation to avoid attributing Legionnaires’ disease cases to an incorrect source of infection

**DOI:** 10.2807/1560-7917.ES.2020.25.34.2001484

**Published:** 2020-08-27

**Authors:** Maria Cristina Rota, Maria Scaturro, Maria Luisa Ricci

**Affiliations:** 1 Department Infectious Diseases, National Institute of Health, Rome, Italy

**Keywords:** *Legionella*, Legionnaires disease, epidemiological and environmental investigation, typing

**To the editor:** We read with interest the recent rapid communication by Palazzolo et al. entitled ‘*Legionella *pneumonia: increased risk after COVID-19 lockdown? Italy, May to June 2020’ [1]. 

The report aimed at describing a Legionnaires’ disease case after COVID-19 lockdown and to underline* “the importance of strict monitoring of water and air systems immediately before reopening business or public sector buildings, and the need to consider *Legionella* infections among the differential diagnosis of respiratory infections after lockdown due to the ongoing COVID-19 pandemic”.*

When a Legionnaires’ disease (LD) case (or an outbreak) occurs, it is necessary to carry out an in-depth epidemiological investigation of the case, using a standardised questionnaire. This allows to generate hypotheses on possible sources of infection. The acquired information guides environmental investigations and sampling, which should be performed. The information about suspected sources also, in turn, enables health authorities to find potential additional cases.

The detection of *Legionella* in environmental samples is indispensable for linking the source of infection to the case and for a risk assessment, to prevent the occurrence of further cases [2,3].

The golden standard for *Legionella* typing, according to the ESCMID Study Group for *Legionella* Infection (ESGLI) is sequence-based typing (SBT), although recently, core genome multilocus sequence typing (cgMLST) and whole genome sequencing (WGS) have been employed by some studies to further discriminate *Legionella* strains with very common sequence types [4,5].

In the paper by Palazzolo et al., an accurate epidemiological investigation aimed at finding all possible sources of infection in addition to the workplace of the case, in the 14 days before the onset of symptoms, was not reported. For example, the study of the patient’s residence and other places possibly attended, visited or passed through (e.g. dentist, fitness centre, streets etc.) is not described, nor for example, irrigation systems that the patient may have used. Furthermore, at the patient’s workplace, no environmental strains were detected. Moreover, the human strain was neither isolated by culture nor detected by PCR on respiratory secretions.

As the authors themselves write: “*Given the plethora of water sources to which a person may be exposed during the incubation period, determining the source of waterborne* Legionella *infection can be very challenging*”**[6].

In fact, it is well known that *Legionella* is a ubiquitous microorganism and the infection can be acquired anywhere. Hence, the article does not demonstrate that LD was acquired in the working place (no *Legionella* were found there) due to the lockdown and concluding this is not justified by the study results.

To demonstrate the source of infection of LD, not only the bacterium must be present in this source (while on the contrary the authors state that *“In the restaurant and in the kitchen where the case worked, there are no air conditioning systems. The staff toilets (shower, sink faucets, sink water and cold water, and hot and cold water bidets) and kitchen taps were sampled and all resulted negative for *Legionella*”),* but according to international scientific literature and ESGLI protocols, it is of paramount importance that sequence types of the human and environmental isolates (both not available in this case) match [3,4].

Notwithstanding, we fully agree that, during the coronavirus disease (COVID)-19 lockdown the use of water of buildings with disused or poorly used water systems may be associated with an increased risk of acquiring *Legionella* infection. For this reason, managers of tourist accommodation sites and buildings in general, have been informed of this risk and should apply adequate prevention and control measures for LD [6,7].

## References

[1] Palazzolo C Maffongelli G D’Abramo A Lepore L Mariano A Vulcano A *Legionella* pneumonia: increased risk after COVID-19 lockdown? Italy, May to June 2020. Euro Surveill. 2020;25(30):2001372. 10.2807/1560-7917.ES.2020.25.30.2001372 32734857 PMC7393852

[2] Legionnaires' disease outbreak investigation toolbox. Available at: https://legionnaires.ecdc.europa.eu/?pid=10

[3] Phin N Parry-Ford F Harrison T Stagg HR Zhang N Kumar K Epidemiology and clinical management of Legionnaires’ disease. Lancet Infect Dis. 2014;14(10):1011-21. 10.1016/S1473-3099(14)70713-3 24970283

[4] Afshar B Fry NK Bellamy W Underwood AP Harrison TG Members of the European Working Group for Legionella Infections External quality assessment of a DNA sequence-based scheme for epidemiological typing of Legionella pneumophila by an international network of laboratories. J Clin Microbiol. 2007;45(10):3251-6. 10.1128/JCM.00898-07 17687012 PMC2045376

[5] Wüthrich D Gautsch S Spieler-Denz R Dubuis O Gaia V Moran-Gilad J Air-conditioner cooling towers as complex reservoirs and continuous source of *Legionella pneumophila* infection evidenced by a genomic analysis study in 2017, Switzerland. Euro Surveill. 2019;24(4):1800192. 10.2807/1560-7917.ES.2019.24.4.1800192 30696527 PMC6351994

[6] ESGLI Guidance for managing *Legionella* in building water systems during the COVID-19 pandemic. Available at: https://www.escmid.org/research_projects/study_groups/study_groups_g_n/legionella_infections/

[7] Ricci ML, Rota MC, Scaturro M, Veschetti E, Lucentini L, Bonadonna L, et al. Guida per la prevenzione della contaminazione da Legionella negli impianti idrici di strutture turistico recettive, e altri edifici ad uso civile e industrial non utilizzati durante la pandemia COVID-19. [Guide for the prevention of Legionella contamination in the water systems of tourist accommodation facilities and other buildings for civil and industrial use, not used during the COVID-19 pandemic]. Rapporto ISS COVID-19 n. 21/2020. Rome: Istituto Superiore di Sanità. 3 May 2020. Italian. [Accessed 7 Jul 2020]. Available from: https://www.iss.it/documents/20126/0/ Rapporto+ISS+COVID-19+21_2020.pdf/15088523-3e22-55e1-d28d-f37d9aafd186?t=1588953957255

